# Fluorosilicone as an Omnimold for Microreplication

**DOI:** 10.3390/mi9080406

**Published:** 2018-08-16

**Authors:** Teng Zhang, Xiaokui Yue, Dan Sameoto

**Affiliations:** 1 Department of Mechanical Engineering, University of Alberta, Edmonton, AB T6G 2G8, Canada; tzhang1@ualberta.ca; 2 National Key Laboratory of Aerospace Flight Dynamics, Northwestern Polytechnical University, Xi’an 710072, China; xkyue@nwpu.edu.cn

**Keywords:** fluorosilicone, soft lithography, molding, adhesion, silicone, polyurethane, casting

## Abstract

Soft lithography and replica molding have been an integral part of polymer basic microfabrication for over 20 years. The use of silicone rubber materials as either molds or directly molded parts are well described in the literature and have provided researchers with an easily accessible technique to reproduce complex micro and nanostructures with minimal costs and technical challenges. Yet, for many applications, the use of standard silicones may not necessarily be the best choice, either as a mold material or as a replicated surface. For those instances where a mold is required that is high temperature tolerant, flexible, durable and capable of being used as a mold for multiple materials including silicone rubber, the most commonly used silicone rubber, Sylgard-184, has substantial deficiencies. In this work, we introduce a new material, Fluorosilicone that has not been described in the microfabrication field in detail and determine it is capable of reproducing micro structures via soft lithography techniques and being used as a mold for thermoplastic and thermosetting polymers, including silicone rubbers. Material compatibility, appropriate processing conditions for quality replicas and demonstration of extremely fast production of silicone microstructures are reported.

## 1. Introduction

In some applications of microfabrication, like microfluidic devices or gecko-like adhesives, fabrication of polymer-based samples via soft lithography including hot embossing, injection molding, or lamination, is widely applied [1,2,3,4]. Compared with standard lithography, many self-lithography processes can be done outside a cleanroom, which reduces the cost and complexity, and large-scale production is possible. The key part in the fabrication process is the production of a high quality negative mold with desired structures [5].

When rigid molds are used, the mold material ought to have some specific properties [5]: (i) High stiffness and strength, (ii) good physical and chemical resistance, (iii) easy to be fabricated with different structures, and (iv) low surface energy for aid in demolding. Generally, there are two kinds of molds—rigid and soft molds. Compared with rigid molds, like SU-8 (MicroChem, Worcester, MA, USA) on Si wafers or Poly methyl methacrylate (PMMA, Plaskolite, Columbus, OH, USA) substrates, soft molds fabricated by Polydimethylsiloxane (PDMS) or other elastomers typically can have longer life cycles, because the thermal or demolding stress does not affect the mold but for rigid ones, structures may be damaged in curing or demolding [6]. Moreover, soft molds permit molding to be completed on non-flat surfaces or be more easily adapted to roller style continuous molding processes. Of the materials that can be used to make soft molds, PDMS is often selected due to its good physical and chemical properties, and its ease of fabrication outside a cleanroom environment, which reduces the time, complexity, and cost [7,8]. However, PDMS has some disadvantages that limit its applications in fabricating soft molds:

- Its tear strength can be low (about 0.87 kN/mm for Dow Corning^®^ Sylgard-184), so it is easy to rip and is challenging to obtain a thin mold;
- When PDMS structural material for the final product is required, surface modification of the mold is required to achieve anti-sticking behavior [6];
- PDMS will swell in some organic solvents [7,9], which makes several types of solvent casting unfeasible.

Fluorosilicone is an elastomer of siloxane polymers with fluorinated organic substituents bonded to silicon, which can be used in extreme environments and may resist damage/deformation when in contact with various chemicals that regular silicone rubbers cannot [10]. According to literature, most research on fluorosilicone is about its composition and fabrication [11,12,13,14,15] and its applications in the fields of fuels, oils, injection molding as O-rings, releasing or coating layers [16,17,18,19,20,21,22]. However, in the field of microelectromechanical systems (MEMS), microfluidics or microfabrication in general, fluorosilicone has not been investigated yet, especially the use of fluorosilicone as a material in soft lithography for durable, flexible and high temperature tolerant molds.

Several important properties of a commonly used PDMS (Dow Corning^®^ Sylgard-184, Midland, MI, USA) and an investigated fluorosilicone (Dow Corning^®^ 5-8601 Fluorosilicone, Midland, MI, USA) are compared in Table 1. They have the same hardness, and PDMS has larger tensile modulus, but the fluorosilicone has a much larger tear strength (about 23 times that of PDMS), and longer pot life at room temperature (96 h vs. 1.6 h). Additionally, fluorosilicone is more resistant to organic solvents [10] which can be a benefit in specific soft lithographic processes like solvent assisted micromolding.

This paper investigates the usage of fluorosilicone in soft lithography in terms of compatibility with a range of master mold materials and castable structural materials from the fluorosilicone. Both unstructured and highly structured dry adhesive molds are used for molding compatibility trials. The master mold materials tested for releasing negative fluorosilicone replicas include; PMMA, SU-8, silicon wafer (Silicon Materials, Glenshaw, PA, USA), glass, polylactic acid (PLA, Filaments.ca, Mississauga, Ontario, Canada), Kraton^®^ G1657 styrene ethylene butylene styrene (SEBS, Kraton, Houston, TX, USA), shape memory polymer (SMP, MM-2520, SMP Technologies Inc, Tokyo, Japan), polystyrene (PS, Fisher Scientific, Hampton, NH, USA), PDMS (BJB Enterprise^®^ TC-5030, BJB Enterprises, Tustin, CA, USA; and Dow Corning^®^ Sylgard-184, Dow Corning). To cast thin fluorosilicone molds on master templates, we use a modified lamination process due to the extremely high viscosity of the uncured fluorosilicone. Lamination can be done at room temperatures for example, while for injection, the mold should be heated to between 170 °C and 220 °C [26]. Once high quality fluorosilicone negative molds are produced, the molding and demolding properties of different thermoplastic and thermosetting polymers on the flat and negative featured fluorosilicone mold are tested. The results show that nearly all the polymers in the experiment can be fully cured and demolded well from the fluorosilicone negative mold. As a demonstration of usefulness of fluorosilicone as a soft lithography mold in production applications, a Sylgard-184 replica of dry adhesive fibers has been cured in as little as a minute without vacuum degassing from a fluorosilicone negative template. This dramatically reduces time and complexity of manufacturing of silicone rubber microstructured surfaces.

## 2. Fabrication Process

**Figure 1:**
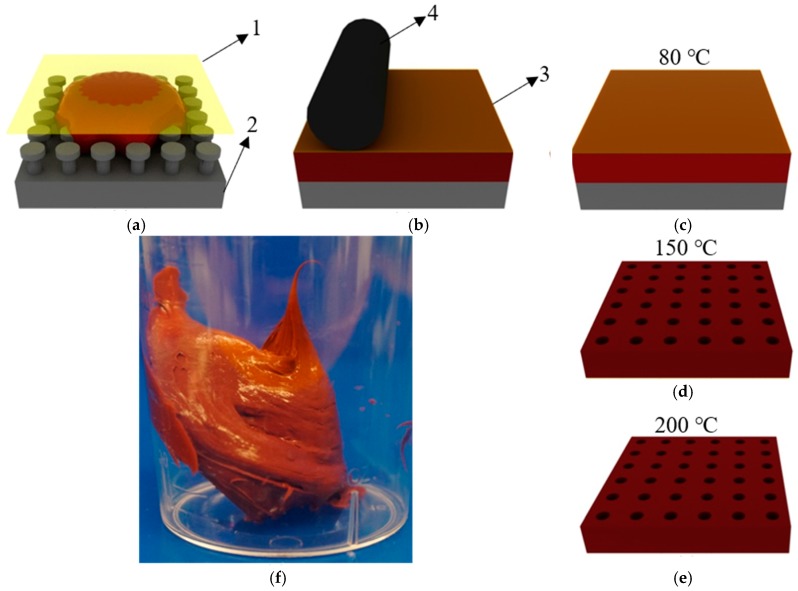
Fabrication process of making fluorosilicone mold. 1: Thin film; 2: mold; 3: fluorosilicone; 4: roller; (**a**) The film is coved on fluorosilicone with the mold; (**b**) Modified lamination; (**c**) Curing at 200 °C; (**d**) Curing at 150 °C after demolding; (**e**) Post-curing at 200 °C after the film is peeled off; (**f**) Photo of fluorosilicone after mixing.

Dow Corning^®^ 5-8601 Fluorosilicone Liquid Silicone Rubber, is designed for liquid injection molding [24] and is used and tested in this work as a representative material because it had the lowest viscosity of the fluorosilicones available to us. The product is supplied as a two-component reaction cure system that is mixed at a 1:1 ratio. Due to the high viscosity of fluorosilicone as shown in Figure 1f, traditional soft lithography methods for making silicone rubber molds cannot be used. Instead, a lamination method, adapted from previous work [27] is applied. The fabrication process after mixing and degassing is shown in Figure 1a–e. A planetary mixer is normally recommended to avoid introducing air bubbles into the viscous fluorosilicone paste but as this equipment was unavailable, a telfon coated steel rod was used to hand mix the material. Then the mixed fluorosilicone is degassed for 24 h, at ~5 kPa, to remove as much air as possible. After that, it is transferred to the mold with a spatula, and a transparency film (3M^®^ PP2910 Plain Paper Copier Transparency Film, The 3M Company, Maplewood, MN, USA) is lightly pressed on the fluorosilicone to provide a relatively flat backing. The whole stack is then placed in a Ziploc bag and then run through a laminator at room temperature with progressively smaller gaps between rollers until a relatively uniform fluorosilicone layer is achieved. Then, the fluorosilicone with the mold and the film is cured at 80 °C for 96 h. The fluorosilicone and the film is demolded from the mold, and they are cured at 150 °C again for 30 min. Finally, after peeling off the film from the fluorosilicone mold, a post-cure is done at 200 °C for at least 30 min.

**Figure 2:**
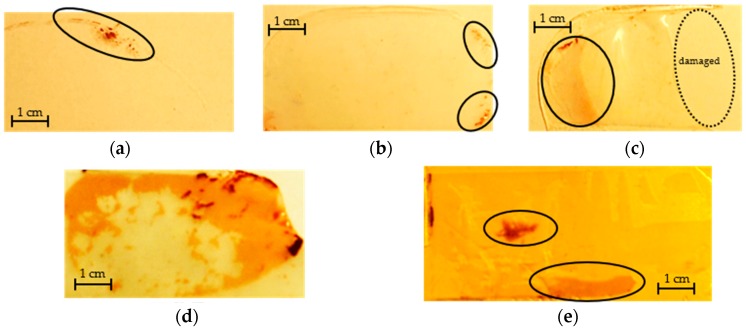
Residuals on the different molds after demolding. In the above figures, except for Figure 2d, the residuals are all circled. In Figure 2c, the dash circle represents the area that is damaged in demolding. (**a**) Glass; (**b**) PLA; (**c**) SEBS; (**d**) SMP; (**e**) Sylgard-184.

In Table 2, residual ratio is defined as the ratio between the area of the residual fluorosilicone after demolding and the area of the mold initially covered by the fluorosilicone. The molds which can be demolded but with residuals after demolding are shown in Figure 2. The scale bars in Figure 2 represent a distance of 1 cm.

For the flat molds, fluorosilicone can be demolded well from the silanized glass, silianized silicon, and PMMA with no residuals observed. It is impossible to demold fluorosilicone from the bare silicon wafer and demolding from glass is possible but leaves substantial residual material as shown in Figure 2a. Generally, silane treatment is beneficial for making the fluorosilicone mold and the fluorosilicone adhesive behavior is similar to PDMS in this respect.

For the positive molds, only SU-8 and PS structural materials show good performance with no residuals being left after fluorosilicone is demolded. For other master molds, some fluorosilicone is left, and in the case of SEBS, the mold is heavily damaged after use.

**Figure 3:**
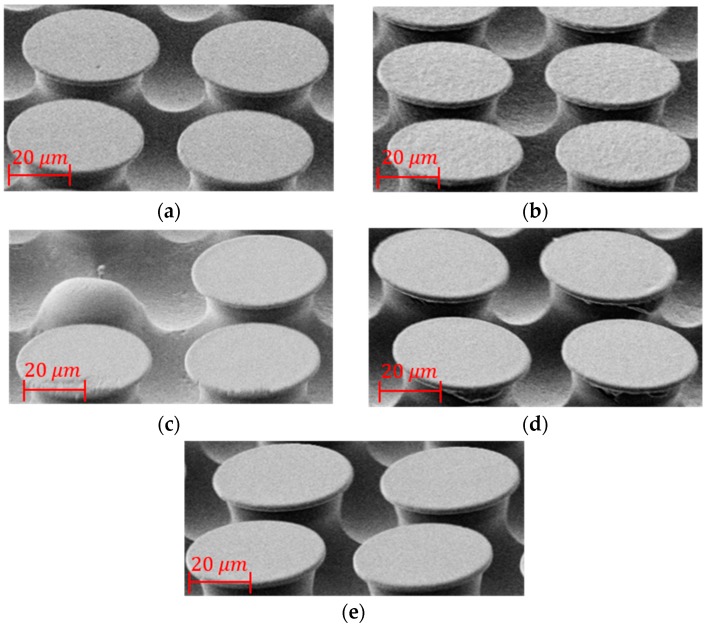
Scanning electron microscope (SEM) samples of polystyrene (PS) replicas from the fluorosilicone mold. (**a**) Duplications from the PS master mold. (**b**) Duplications from the PLA master mold. (**c**) Duplications from the SEBS master mold. (**d**) Duplications from the SMP master mold. (**e**) Duplications from the Sylgard-184 master mold.

To examine the microscale fidelity of the fluorosilicone negative molds, the corresponding PS duplications from PS, PLA, SEBS, SMP, and Sylgard-184 molds are fabricated by compression molding and SEM images of typical features are shown in Figure 3. Because fluorosilicone is left on some parts of the molds with the exception of the PS positive, only the areas of fluorosilicone that cleanly demolded are used for making the imaging replicas.

In Figure 3, all the scale bars represent 20 μm. For the duplication from the PS mold, the fibers are high quality, and the surfaces are smooth as in Figure 3a. In contrast with the SU-8 mold, the PS mold can be fabricated by hot embossing outside the cleanroom, which decreases the time consumption and cost and shows good potential for fast and massive production. For the duplication from the PLA mold, the fibers look acceptable, but the top surfaces are rough as seen in Figure 3b. For the replicas from the SEBS and SMP master mold in Figure 3c,d, the fibers look damaged, even though they are from good areas of the mold. While for duplications from the Sylgard-184 master mold, the fibers look the same quality as the fibers from the PS master mold as shown in Figure 3a,e. For Sylgard-184 and TC-5030, both of them are PDMS, but they show different performance. Fluorosilicone mold can be demolded from the master Sylgard-184 mold although some residuals are left, but fluorosilicone is bonded permanently with TC-5030 after it is fully cured.

## 3. Fluorosilicone as a Negative Mold for Soft-Lithography Replication

After the high quality fluorosilicone mold is fabricated, its usefulness as a negative mold for replicating different structural materials is tested. Many thermosetting and thermoplastic rubbers and hard polymers used by our group are tested for compatibility with fluorosilicone molds, including Dow Corning^®^ 93-500 (Dow Corning), ECO-Flex™ 00-10 (Smooth-on, Macungie, PA, USA), Dow Corning 7-9850 (Dow Corning), Dragon skin^®^ 30 (Smooth-on,), Dow Corning^®^ MS-1002 (Dow Corning), polyurethane (PU, Normac^®^ NR-906, Normac Adhesive Products Inc., Burlington, ON, Canada), Chronoprene 5A (AdvanSource^®^ biomaterials, Wilmington, MA, USA), PS (Fisher Scientific), SEBS (Kraton^®^ G1657), SMP (SMP Technologies Inc), PDMS (TC-5030, and Sylgard-184), 3M™ ESPE™ Imprint™ 3 Regular Body (The 3M Company) and Momentive RTV 159 silicone adhesive (Momentive, New York, NJ, USA).

**Figure 4:**
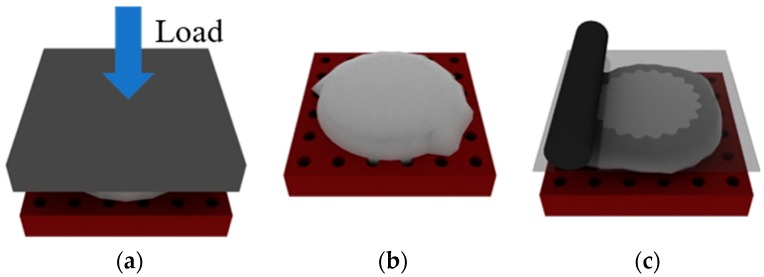
Fabrication processes used to produce polymer samples from fluorosilicone molds. (**a)** Hot embossing; (**b**) vacuum; and (**c**) lamination.

All these materials have specific processing conditions and methods that are best suited for their replication from fluorosilicone molds, but manufacturing techniques used fall under compression molding, vacuum casting and lamination as shown in Figure 4. Compression molding was completed for thermoplastics and thermoplastic elastomers at 220 °C, with a load of approximately 0.5 MPa using a process described previously for silicone rubber molds [28]. Vacuum casting was done with thermosetting materials that had relatively low viscosities and was done according to material recommendations, and lamination was completed for thermosetting materials that cured exceptionally fast or had viscosities too high to work with vacuum casting. Some important properties of the materials used in these experiments are listed in Table 3.

The qualitative results on demolding yields are listed in Table 4. For Sylgard-184, due to its low tear strength, a Kapton^®^ polyimide tape (DuPont, Wilmington, DE, USA) with silicone adhesive is gently placed on the uncured PDMS after vacuum degassing. After being fully-cured, the Sylgard-184 sample with the tape is demolded together from the mold. 

All the thermosetting polymers in the experiment can be cured with no evidence that the fluorosilicone inhibits the cure in any way. For the very soft materials, the fibers are damaged when being demolded from the negative mold. Some of them, including Dow Corning^®^ 93-500 and Dow Corning^®^ MG 7-9850, are so soft that they are difficult to be demolded even from the flat mold, but they are fully cured and can be demolded without leaving residue. Chronoprene 5A and ECO-Flex™ 00-10, do not pose difficulties to demold from the flat mold. For the polymers which are stiffer than Ecoflex 00-10, almost all of them can be demolded from both the flat and negative mold with the exception of RTV 159 adhesive. which is used as a high strength silicone rubber sealant. So, it is not strange to see that it is bonded strongly to the negative mold.

**Figure 5:**
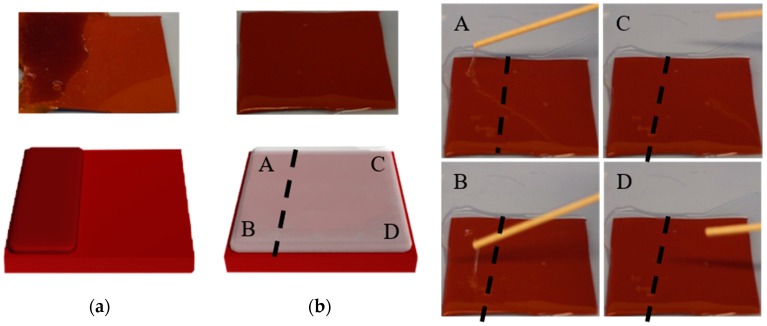
Experiment of PU on the fluorosilicone mold (the dash line represents the edge of PU, on the left of the line, PU is cured on and demolded from the mold). Left: (**a**) PU on the part of the mold, (**b**) Sylgard-184 on the whole mold after PU is demolded; Right: The photos after the applicator contacts to the different areas as in (**b**)**.**

Ideally, a fluorosilicone mold would work with every material that demolds from it without issue, but unfortunately other factors can play a role. For example, addition curable PDMS like Sylgard-184 cannot be cured on soft polyurethane molds because of inhibition of the crosslinking agent in PDMS by the polyurethane. While fluorosilicone molds work for both polyurethane and Sylgard-184, the same mold was found to not be compatible with each due to residual PU diffusing into the fluorosilicone. This was confirmed by running the experiment shown in Figure 5. PU is cast on part of a fluorosilicone mold, cured and then demolded. After demolding the PU, Sylgard-184 is cast on the whole mold. The Sylgard-184 is cured at 80 °C for 2 h and then examined by probing with an applicator stick. Touching the PDMS demonstrated that the material was still liquid on the portion of the mold previously covered with PU (areas A and B), and solid everywhere else (areas C and D). The video (Video S1) shows the results.

## 4. Discussion

**Figure 6:**
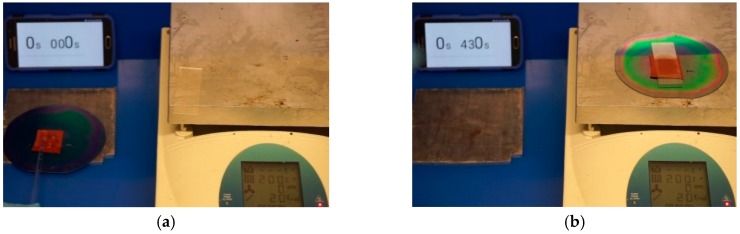

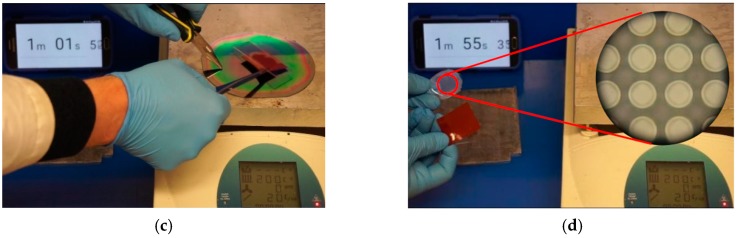
Flash curing of PDMS samples via fluorosilicone mold: (**a**) Sylgard-184 is rubbed on the fluorosilicone mold; (**b**) Sylagrd-184 is heated at 200 °C; (**c**) the sample is removed after 1 min; (**d**) the sample is demolded and inspected.

One of the limitations to fast and inexpensive processes using soft lithography is the time to cure the silicones (often an hour or more for many applications). The higher temperature tolerances and durability of fluorosilicone offer a way around this limitation. Fluorosilicone molds can work at up to 225 °C [24] in dry environments, so a trial to determine the feasibility of quick curing Sylgard-184 at 200 °C was completed. The soft mold is damage resistant, so PDMS can be spread into the holes with a light pressure applied by a glass rod and microscale fibers are replicated without the need for vacuum degassing. A video recording of the trial is shown in the supplementary information (Video S2) and some key points of the video are shown in Figure 6. After mixing and degassing, the liquid PDMS is gently rubbed onto a fluorosilicone mold and then transferred to a hotplate held at 200 °C. The sample was solidified within a minute and then the mold is removed from the hot plate. The sample cools to room temperature within 30 s, at which point the sample is demolded and inspected under a microscope. In total, the time from dispensing to completion is approximately 2 min. For Sylgard-184, the sample should normally be cured for over 48 h at room temperature or 2 h at 80 °C. The high temperature tolerance and mechanical durability of a fluorosilicone mold permits silicone casting to be completed orders of magnitude more quickly than traditional molding, and because fluorosilicone mold is soft and flexible it can be used to thermal roll to roll nanoprinting system for massive production, as in Reference [29].

## 5. Conclusions

Fluorosilicone has been introduced as a mold material for soft lithography. Compared with rigid molds, soft molds can be more mechanically durable, and this particular material is usable with a very wide range of castable and thermally moldable materials. While more challenging to produce high quality initial templates from lithographically patterned molds, the high temperature tolerance, and good mechanical durability of fluorosilicone makes it an excellent choice for fast casting of commonly used silicone rubber materials like Sylgard-184. Light mechanical pressure can be used to fill in arrays of gecko-inspired adhesive fibers and the flexible nature of fluorosilicone means that continuous manufacturing of micro or nanostructured PDMS surfaces may be commercially viable in roll-to-roll processes because curing can be accelerated by orders of magnitude when using fluorosilicone as a mold material.

## Figures and Tables

**Table 1:** Comparison of Sylgard-184 and 5-8601 fluorosilicone.

Property	Unit	Materials	
		PDMS	Fluorosilicone
Elongation	%	120	350
Tear strength	kN/mm	0.87	20
Tensile modulus	MPa	7.1	6.2
Durometer Shore A	points	44	45
Pot life at room temperature	hours	1.4	96

Note: The data of Dow Corning^®^ Sylgard-184 and Dow Corning^®^ 5-8601 fluorosilicone in References [23,24] are compared here. More details about the Dow Corning^®^ 5-8601 fluorosilicone can be found in References [25,26].

**Table 2:** Results of demolding fluorosilicone from different molds.

Materials	Mold Category	Ease of Demolding	Residual Ratio	Notes
PMMA	Flat	++	Not observable	Easy to demold, works well
Silanized glass	Flat	++	Not observable	Easy to demold, works well
Glass	Flat	-	~5%	Residual fluorosilicone on the glass
Silanized silicon wafer	Flat	++	Not observable	Easy to demold, works well
Silicon wafer	Flat	--	100%	Impossible to demold
SU-8	Structured	++	Not observable	Easy to demold, works well
PLA	Structured	+	~5%	The top surface of the PLA looks rough after demolding
SEBS	Structured	--	~40%	SEBS mold is damaged in demolding
Polystyrene	Structured	++	Not observable	Easy to demold, works well
SMP	Structured	-	~50%	Residual fluorosilicone on the SMP mold
TC-5030	Structured	--	100%	Impossible to demold
Sylgard-184	Structured	-	~30%	Residual PDMS on the fluorosilicone mold

**++.** very easy; +: easy; –: difficult; --: Impossible; PLA = polylactic acid; SEBS = styrene ethylene butylene styrene; SMP = shape memory polymer.

**Table 3:** Properties of materials used for replicas from fluorosilicone masters.

Polymers	Type	Fabrication Method	Hardness	Link
Chronoprene 5A	Thermoplastic	C	5 A	http://www.advbiomaterials.com/products/elastomeric/chronoprene.html
Dow Corning 93-500	Silicone rubber	V	46 A	http://www.centralcoating.com/wp-content/uploads/2014/11/93-500.pdf
ECO-Flex	Silicone rubber	V	00–10	https://www.smooth-on.com/tb/files/ECOFLEX_SERIES_TB.pdf
Dow Corning^®^ MG 7-9850	Silicone rubber	V	N/A	http://www.healthcare-plus.com.tw/big5/pdf/02-05.pdf
PS	Thermoplastic	C	N/A	N/A
SEBS	Thermoplastic	C	47 A	http://www.talasonline.com/images/PDF/ProductDataSheet/Kraton_G1657_datasheet.pdf
SMP	Thermoplastic	C	30 D	http://www2.smptechno.com/en/smp/
PU	Thermoset	V	85–90 A	http://www.normacadhesives.com/fr/normac-products/castable/nr-906.html
Sylgard-184	Silicone rubber	V	43 A	https://consumer.dow.com/content/dam/dcc/documents/en-us/productdatasheet/11/11-31/11-3184-sylgard-184-elastomer.pdf?iframe=true
TC-5030	Silicone rubber	V	25–35 A	https://bjbenterprises.com/media/wysiwyg/pdfs/Silicones/TC-5030.pdf
Dragon Skin^®^ 30	Silicone rubber	V	30 A	https://www.smooth-on.com/tb/files/DRAGON_SKIN_SERIES_TB.pdf
MS-1002	Silicone rubber	V	74 A	https://www.protolabs.co.uk/media/751887/dow_corning_electronics_ft_ms-1002.pdf
3 Regular Body	Silicone rubber	L	55 A	http://multimedia.3m.com/mws/media/391881O/imprint-3-vps-impression-materials.pdf
RTV 159 adhesive	Silicone rubber	L	28 A	https://www.momentive.com/en-us/products/tds/rtv157-and-rtv159/

C = compression molding; V = vacuum; L = lamination; PU = polyurethane.

**Table 4:** Results of peeling polymers from fluorosilicone molds after being cured.

Polymers	Flat Mold	Negative Mold	Note
Chronoprene 5A	Work	Too soft to form stable structures	Not difficult to demold
Dow Corning^®^ 93-500	Work	Too soft to form stable structures	Difficult to demold even for the flat mold, although it is cured well
ECO-Flex™ 00-10	Work	Too soft to form stable structures	The same as Chronoprene 5A
MG 7-9850	Work	Too soft to form stable structures	The same as Dow Corning^®^ 93-500
PS	Work	Work	Works well and very easy to demold
SEBS	Work	Work	The same quality as PS
SMP	Work	Work	The same quality as PS, although more difficult to compression mold
PU	Work	Work	The same as PS
Sylgard-184	Work	Work	Kapton polyimide tape is needed to demold without tearing. Good quality samples can be obtained with tape or with thicker PDMS
TC-5030	Work	Work	When cured too long, the fibers are damaged after peeling off
Dragon Skin^®^ 30	Work	Work	Good performance when being cured at room temperature
MS-1002	Work	Work	Good performance, but due to its low tear strength, easy to be broken
3 Regular Body	Work	Work	Good performance
RTV 159 adhesive	Work	Failed	Good performance for the flat mold, but failed to negative mold

## Supplementary Materials

The following are available online at http://www.mdpi.com/2072-666X/9/8/406/s1. Video S1: Experiment of PU on the fluorosilicone mold. Video S2: Flash curing of PDMS samples via fluorosilicone mold.
